# Particulate Matter: The Clot Thickens: New Clues to PM Action

**DOI:** 10.1289/ehp.115-a580b

**Published:** 2007-12

**Authors:** Adrian Burton

Particulate matter (PM), largely sooty particles from the exhausts of diesel vehicles and fossil fuel–burning power stations, has long been associated with increased cardiovascular events, with peaks occurring within 24 hours of spikes in ambient PM concentration. Researchers, however, have not been sure why this is so. A new report in the October 2007 issue of the *Journal of Clinical Investigation* shows that the increase in cardiovascular events seen after spikes in PM pollution may be due to macrophages in the irritated lungs producing increased amounts of interleukin-6 (IL-6), a cytokine that promotes clotting. This increases the chances of suffering a heart attack or stroke, especially in individuals already at risk due to lung disease or atherosclerosis.

“This has been a long-standing question,” says first author Gökhan Mutlu, an assistant professor of pulmonary and critical care medicine at Northwestern University. “These particles, once inhaled, cause inflammation of the lungs, but how this is connected to cardiovascular events was unclear.”

To study the effects of PM exposure, the researchers intratracheally administered a solution of PM_10_ (particles less than 10 μm in diameter commonly found in polluted city air) to mice. According to the article, the 10-μg dose administered is the equivalent of that to which a person would be exposed when ambient PM_10_ concentrations hit a moderate elevation of 150 μg/m^3^.

During this work the team noticed that incisions made in animals exposed to PM_10_ 24 hours earlier bled far less than did those in unexposed mice; exposure seemed to speed up clotting times. Tests showed that exposed mice had an increased number of platelets, reduced prothrombin and activated partial thromboplastin times, a higher concentration of plasma fibrinogen, and increased levels of blood factors II, VIII, and X—conditions that are all consistent with increased and quicker clotting. In addition, plasma thrombin–antithrombin III complex levels were four times higher in the exposed mice, further suggesting that exposure promotes intravascular thrombosis.

“Since IL-6 increases the transcription of procoagulant proteins, we checked its concentration in the bronchoalveolar fluid of exposed mice and found it to be increased sixteenfold,” explains Mutlu. “[This IL-6] seemed to be produced by the increased number of macrophages in the lungs of these animals; when we depleted [these macrophages] with liposomal clodonate, the IL-6 level was not raised. Moreover, when we exposed transgenic mice that do not produce IL-6, their clotting times were not shortened. All this indicates that irritation of the lungs causes an increase in the number of macrophages and IL-6 secretion, which makes blood clot more easily, raising the chances of suffering a stroke or heart attack.”

Particles smaller than PM_10_ that are also common constituents of diesel-polluted air may have similar effects, Mutlu adds.

Although no increase in IL-6 was seen in the control mice, some experts think that the model may not exactly reflect what happens in humans. Terry Tetley, a professor of lung cell biology at the National Heart and Lung Institute, Imperial College, London, explains it is not clear how the instilled dose of PM_10_ in the study relates to real-world doses retained in the human lung, a significant percentage of which is exhaled.

William MacNee, clinical director of the Edinburgh Lung and the Environment Group Initiative at the Colt Laboratory, United Kingdom, agrees: “The authors’ argument that the ten-microgram dose instilled is a ‘moderate’ dose that in some way relates to human inhalation exposure is arguable. In fact [this represents] a very large dose in a mouse, delivered instantaneously at a high dose rate by instillation. Contrast the dramatic effects seen here with the very minor effects in measurable coagulant parameters [reported in other studies] following actual inhalation of concentrated ambient particles.”

Both experts agree, however, that IL-6 may certainly be involved in PM-induced deaths, although this remains to be proven. Unfortunately for the man in the street, avoiding exposure to elevated PM_10_ may be the only way to prevent its irritating effects. Says Tetley, “At present, it is unclear whether predicting air pollution episodes and using strategies such as taking aspirin or wearing face masks if cycling through heavy traffic will be realistic or even useful as we don’t know the exact mechanisms involved or how to deal with the consequences. Possibly the best strategy is to reduce health effects by reducing air pollution.”

